# Phytoplankton phenology through gene expression during the North Atlantic spring bloom decline

**DOI:** 10.1093/ismeco/ycag179

**Published:** 2026-06-26

**Authors:** Meredith G Meyer, Olivia Torano, Natalia L Llopis-Monferrer, Nicolas Cassar, Melanie R Cohn, Mark A Brzezinski, Adrian Marchetti

**Affiliations:** Department of Earth, Marine, and Environmental Sciences, University of North Carolina at Chapel Hill, Chapel Hill, NC 27514, United States; Department of Earth, Marine, and Environmental Sciences, University of North Carolina at Chapel Hill, Chapel Hill, NC 27514, United States; Monterey Bay Aquarium, Research Institute, Moss Landing, CA 95039, United States; UMR7144 Adaptation and Diversity in Marine Environment (AD2M) Laboratory, Ecology of Marine Plankton team, Station Biologique de Roscoff, Sorbonne University, CNRS, Roscoff, France; Division of Earth and Climate Sciences, Nicholas School of the Environment, Duke University, Durham, NC 27708, United States; Department of Earth, Marine, and Environmental Sciences, University of North Carolina at Chapel Hill, Chapel Hill, NC 27514, United States; Department of Ecology, Evolution and Marine Biology, Marine Science Institute, University of California, Santa Barbara, CA 93106, United States; Department of Earth, Marine, and Environmental Sciences, University of North Carolina at Chapel Hill, Chapel Hill, NC 27514, United States

**Keywords:** phytoplankton, spring bloom, metatranscriptomics, North Atlantic

## Abstract

While phytoplankton dynamics in the annual North Atlantic spring bloom have been well characterized, the physiological underpinnings driving these changes and their net impact on the biogeochemistry of the region are less understood. Phytoplankton metabolism is both affected by and influences the region’s nutrient cycling, primary production, and ultimately, the fate of carbon export. Thus, developing an understanding of these processes is critical. Phytoplankton biomass, biological rates, and gene expression data along with associated environmental parameters were measured as part of the NASA EXport Processes in the Ocean from RemoTe Sensing program’s campaign to the North Atlantic to evaluate the relationships amongst these processes within the four most dominant phytoplankton groups (diatoms, dinoflagellates, haptophytes, and chlorophytes) during the spring bloom. We observe a transition from a period dominated by active diatom growth (defined as Phase I) to a period dominated by non-diatom phytoplankton groups (Phase II). Silicic acid depletion appears to limit overall production and reduce competition from diatoms, likely leading to enhanced contributions of haptophytes in Phase II. Expression of key protein-encoding genes involved in cell maintenance, photosynthesis, and nitrogen and vitamin metabolisms varied amongst the taxa throughout the observation period. Expression patterns of diatom genes involved in silicon transport are uncoupled from those involved in nitrate assimilation and photosynthesis, suggesting a change in growth that may be uncoupled from silicification. Our analysis demonstrates how variable silicic acid concentrations can have a significant effect on phytoplankton community structure and regulate primary production, aiding in overall bloom decline.

## Introduction

Given their substantial contribution to seasonal carbon export, spring blooms in the North Atlantic have been extensively studied [1–3], with particular focus on drivers such as physical processes, nutrient concentrations, phytoplankton community composition, and seeding of benthic communities [4–7]. However, substantially less focus has been paid to the specific physiological mechanisms that drive phytoplankton bloom succession and phenology and how these mechanisms are simultaneously linked to the physical and biological drivers of community composition, and the net impacts on biogeochemical cycling during the bloom period. Recent studies have shown that the oceans are experiencing a new form of succession, a taxonomic shift in phytoplankton community composition toward smaller-celled groups, making the oceans overall “greener” (i.e. a shift toward predominance of green algal species; [8, 9]). Therefore, a better understanding of how differences in physiology between phytoplankton groups in response to environmental dynamics impacts net changes in energy flow and carbon cycling is critical for predicting ecosystem functioning and biogeochemical processes.

The annual North Atlantic Spring bloom appears to be initiated in late winter to early spring when a combination of increased irradiance and fluctuating mixed layer depths (MLDs) entrain nutrients from depth and recouple plankton populations in space, causing increased phytoplankton growth and production beginning around March/April [4, 5, 10]. The dominant phytoplankton group during the initiation of the bloom is frequently diatoms, which hold a competitive advantage due to their enhanced nutrient acquisition and storage abilities and fast growth rates under high nutrient conditions [11, 12]. As the bloom begins to decline, the phytoplankton community may shift towards dinoflagellates and haptophytes as previously observed [7, 13–15]. However, recent studies have shown that the proportion of diatoms does not decline as much as previously thought, despite changes in their rates of primary production [16].

Physiological differences between phytoplankton groups make relating taxonomy to physical and biogeochemical parameters important for our understanding of large-scale ecosystem dynamics. Key differences exist in the traits among diatoms, dinoflagellates, and haptophytes, including nutrient requirements, stoichiometry, and metabolism [17]. In addition to all autotrophs’ requirement for carbon, nitrogen, phosphorus, and micronutrients, diatoms have a cellular requirement for silicic acid, and many abundant haptophytes (coccolithophores) require calcium carbonate, creating additional constraints on growth and primary production. Diatoms and haptophytes have been shown to primarily engage in new production (i.e. primary production that utilizes “new” nitrogen sources to the euphotic zone such as nitrate and dinitrogen gas; [18]). This is believed to support shorter food webs [19] and promote enhanced carbon export efficiency [20]. However, all phytoplankton groups have varying cell sizes, carbon content, sinking rates, photosynthetic efficiencies, and mortality rates, undoubtedly impacting their contribution to carbon export and sinking flux.

Here, we examine changes in environmental conditions that drive taxonomic and inferred physiological shifts during the decline phase of the North Atlantic spring bloom at the molecular level and the manner in which these changes relate to net bloom and biogeochemical changes during the NASA EXport Processes in the Ocean from RemoTe Sensing (EXPORTS) campaign to the Porcupine Abyssal Plain (PAP) region in the North Atlantic. The EXPORTS campaign was initiated to compare and contrast the drivers and mechanisms of carbon export in a low productivity, low export region (Ocean Station Papa; [21]) in the subarctic North Pacific to a high productivity, high export region in the North Atlantic (PAP; [3]). This study focuses on the campaign to the PAP region in May of 2021, where an anticyclonic eddy was occupied for 31 days. The comprehensive nature of the program enabled the integration of phytoplankton transcriptomic data with nutrient concentrations, rates of primary production, phytoplankton community composition within the mixed layer, and estimates of carbon export. This allowed us to evaluate how phytoplankton metabolic processes within distinct groups, as inferred through their gene expression, changed during the bloom decline.

## Materials and methods

### Sampling strategy

**Figure 1:**
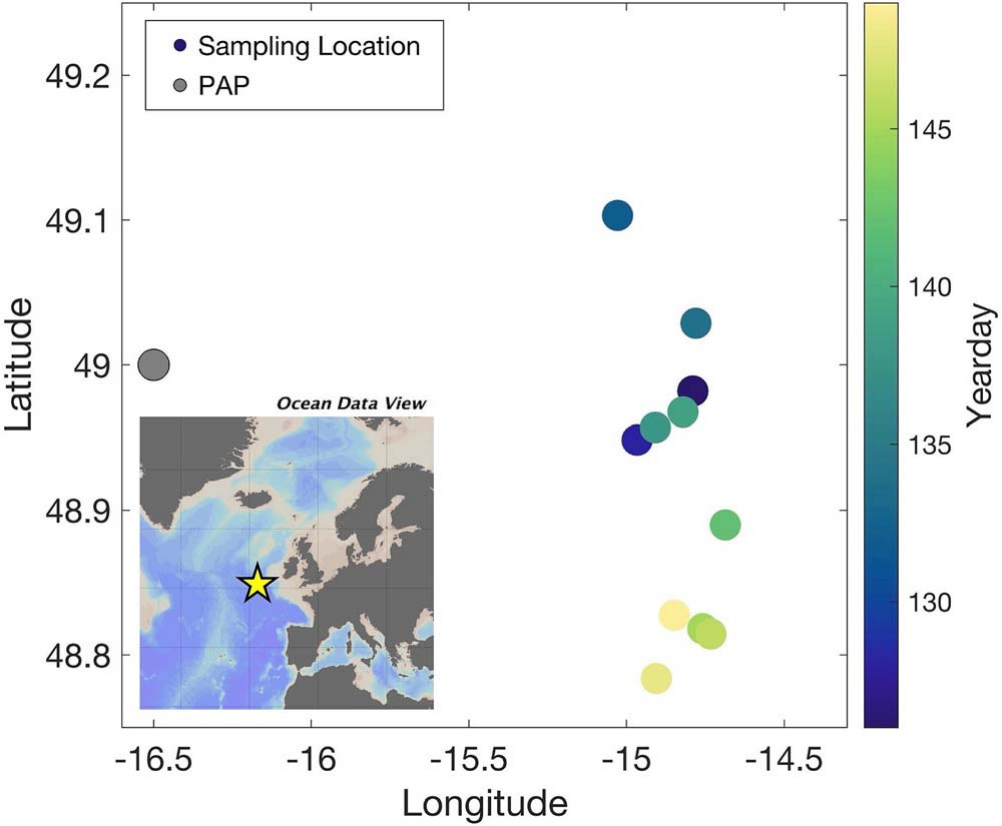
EXPORTS productivity sampling sites in the North Atlantic Ocean. Map of sample locations during the North Atlantic EXPORTS field campaign collected aboard RRS James Cook. Symbol color corresponds to yearday of sampling. The Porcupine Abyssal Plain site is noted in gray. The locations of the PAP site and our sample sites in relation to the European coast are indicated by the star in the inset map.

Samples for primary production, phytoplankton biomass, environmental parameters, and RNA were collected between Yeardays (YDs) 126–148 (6 May to 28 May) in 2021 within the vicinity of the PAP site (49.0°N, 16.5°W) in the North Atlantic Ocean (Fig. 1). Sample collection occurred aboard the Royal Research Ship (RRS) James Cook. The ship sampled in a quasi-lagrangian pattern according to temperature and salinity classifications from a profiling float drogued at 100 m. MLDs were determined according to the 0.03 kg m^−3^ density differential of CTD casts aboard the ship. Samples for dissolved inorganic nutrients, including nitrate (NO_3_^−^), phosphate (PO_4_^3−^), and silicic acid (Si(OH)_4_) and biogenic silica (BSi) were collected throughout the euphotic zone as according to [16] and [22]. For a more detailed discussion of MLD delineation and sampling schemes, see [3] and [16]. All production, biomass, and environmental data have been deposited in the SeaWiFS Bio-optical Archive and Storage System (SeaBASS; https://seabass.gsfc.nasa.gov/cruise/EXPORTSNA/). Raw sequences are deposited at the National Center for Biotechnology Information (NCBI; Table S1).

### Phytoplankton biomass

Triplicate 400 ml bottles were collected for chlorophyll *a* (Chl *a*) concentrations from the depths corresponding to those for the primary production experiments (see below). Bottles were size-fractionated as according to [16], resulting in a < 5 μm size-fraction (small cells) and a ≥ 5 μm size-fraction (large cells) that were summed to produce a total estimate. Filters were immediately frozen at −20°C. Samples were run on a Turner 10 AU fluorometer aboard the ship via the method described in [23]. Estimates of total and size-fractionated (<5 μm, ≥5 μm) particulate carbon (PC) and particulate nitrogen (PN) were provided from the primary production samples [16].

### Primary production

On days when the MLD was deeper than the euphotic zone (defined as the 1% irradiance (I_o_) level), 1 l bottles were collected from five depths corresponding to 65%, 20%, 10%, 5%, and 1% I_o_. On days when the MLD was shallower than the euphotic zone, the 1 l bottles sampled were confined to the I_o_ levels within the mixed layer, which was typically 2–4 depths. Triplicate bottles were inoculated with 180 μmol l^−1^ of NaH^13^CO_3_^−^ isotope, approximating an 8% addition relative to ambient dissolved inorganic carbon (DIC) concentrations and 0.2–0.7 μmol l^−1^ of Na^15^NO_3_^−^ isotope, approximating 16% ambient NO_3_^−^ concentrations (targeted as <10% of ambient concentrations). Bottles were incubated in on-deck, flow-through incubators at their respective light levels for 24 hours before filtration. Estimates of net primary production (NPP) were made from DIC uptake rates [24–26], whereas ^15^NO_3_^−^ uptake rates were converted to carbon units via the Redfield ratio and used to approximate new production [16, 27]. Triplicate bottles provide size-fractionated (<5 μm, ≥5 μm, and total) samples. Estimates of siliceous phytoplankton-specific contributions to total NPP were estimated via silicon-32 uptake (ρSi) according to [22]. Integrated mixed layer values were calculated according to trapezoidal integration [16].

### RNA collection and extraction

Seawater was collected in triplicate, producing 30 samples, from the near-surface (~5 m) using Niskin bottles mounted on a standard CTD rosette (Table S1). After collection, seawater was immediately pumped via low peristaltic pressure for a maximum of 40 minutes through a 0.8 μm Supor filter, resulting in an average of 6 l being filtered. RNA filters were placed into cryovials, flash frozen in liquid nitrogen, and stored at −80°C until onshore extraction.

RNA was extracted using the Qiagen RNAqueous-4PCR plant tissue extraction kit according to the manufacturer's instructions with modifications as follows to enable extraction from 142 mm filters: filters were cut into small pieces into a 50 ml centrifuge tube. Glass beads (0.5 ml) and 7 ml of lysis buffer were then added and vortexed on high speed. Filter pieces were removed, and the lysis buffer was passed through a filter column and eluted. Extracted RNA was treated with DNAse, treated via an RNeasy MinElute Cleanup kit, and stored at −80°C until sequencing. Extracted RNA was sent to Azenta Life Sciences for sequence library preparation and sequencing. mRNA sequence libraries were generated via poly-A selection (Supplemental Methods). Sequencing was performed via Illumina HiSeq on two lanes with ~350 M raw paired-end reads per lane and single index, 2× 150 bp per lane.

### RNA sequencing and metatranscriptomic analysis

Paired-end reads underwent trimming via TrimGalore (v.0.6.2; Altos Labs) to remove sequence adapters and poly-A tails followed by FastQC (v.0.11.9) for quality control, removing any reads that were deemed of too low quality for the analysis (quality score <20 or <50 bp). Trinity (v.2.8.6) was used for de novo assembly of reads according to individual sample day. Following Trinity assembly, cdhit (v.4.8.1; [28]) was used to combine individual contig assemblies into a singular grand assembly of 4 897 877 contigs ≥500 bp (13% annotation coverage; [26]). The grand assembly was annotated, blasting via Diamond, for best-hit functional annotation (e-value cutoff = 10^−5^) via the Kyoto Encyclopedia of Genes and Genomes (KEGG; Release 88.2) and taxonomic annotation via PhyloDB (github.com/allenlab/PhyloDB). Additional, manual blasting of the grand assembly for certain sentinel genes of interest occurred. Fasta files for 41 silicon metabolism-related genes of interest are described in (Table S2; [29–31]). End-to-end read alignment occurred via Salmon (v.10.9.1; [32]) with aligned files and annotated grand assemblies being combined via tximport [33] and resulting in read counts per million (CPM). Code is available at [34].

Two versions of the dataset were visualized. The first version includes gene CPMs normalized (via DESeq2 v.1.50.2) to the datasets first separated into four dominant phytoplankton taxonomic groups (diatoms, dinoflagellates, haptophytes, and chlorophytes). This normalization provided an examination of the gene expression changes within a taxonomic group throughout the observation period. The second version had CPMs normalized to the whole community dataset, providing a community-wide comparison of changes in gene expression among the taxonomic groups throughout the observation period. Additionally, physiologically useful ratios of gene CPMs and rates were evaluated.

## Results

### Nutrient depletion induces a decline in primary production

**Figure 2:**
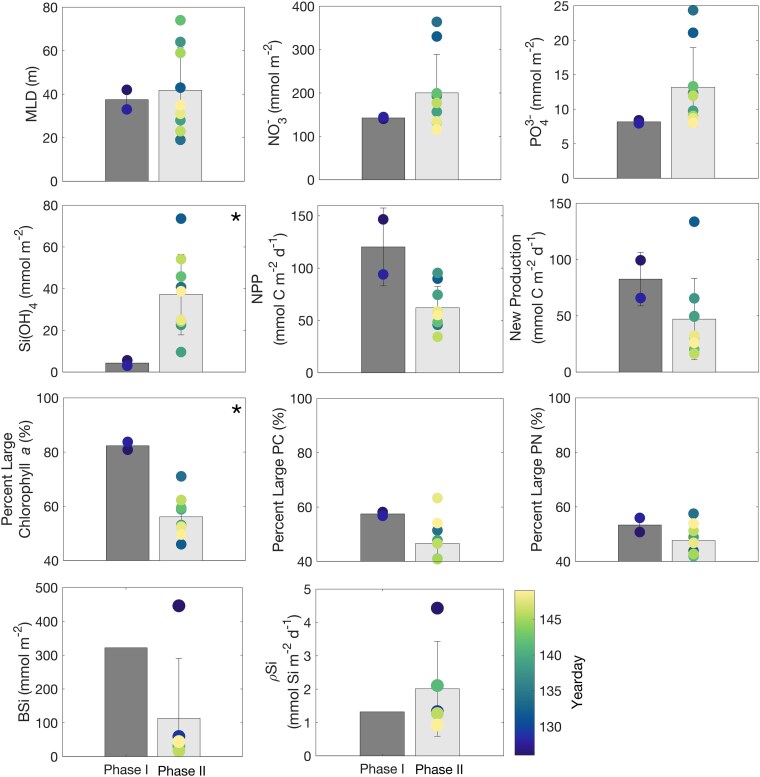
Bar plots of average mixed layer depth (MLD; m) and mixed layer integrated nitrate concentration (NO_3_^−^; mmol m^−2^), phosphate concentration (PO_4_^3−^, mmol m^−2^), silicic acid concentration (Si(OH)_4_; mmol m^−2^), NPP (mmol C m^−2^ d^−1^), new production (mmol C m^−2^ d^−1^), and relative contribution of large phytoplankton (>5 μm) to chlorophyll *a* (Chl *a;* %), PC (%) and PN (%), biogenic silica (BSi; mmol m^−2^), and silicic acid uptake (ρSi; mmol Si m^−2^ d^−1^) in Phase I (dark grey bars) vs. Phase II (light grey bars). Error bars correspond to standard deviations per sample phase. Symbol colors correspond to yearday. Asterisks indicate a statistically significant relationship based on Wilcoxon rank sum testing.

Sampling days were separated into Phase I (PI; YDs 126 and 128) and Phase II (PII; YDs 132–149) based on the RNA patterns, presumably resulting from the impact of a storm event that occurred from YDs 127–130 and substantially altered the properties of the site, including MLD, nutrient inventory, and phytoplankton dynamics [3, 16]. Two additional storms occurred, but the first storm had the largest impact on mixed layer properties. This separation is supported by the averaged differences by phase, where phase-averages in MLD, nutrient concentrations (NO_3_^−^, PO_4_^3−^, Si(OH)_4_), NPP, new production, proxies for the contribution of the large size-fraction to phytoplankton biomass (Chl *a*, PC, PN), BSi, and ρSi show substantial differences (Fig. 2). Partially due to the higher number of samples (8 in PII vs. 2 in PI), variability within PII environmental (and transcriptomic) datasets is much larger, as exemplified by the larger variability and variety of patterns between data points, and influence the statistical robustness of our results (Fig. 2). Overall, macronutrient concentrations are higher (28.9%, 38.0%, 88.4% for NO_3_^−^, PO_4_^3−^, and Si(OH)_4_, respectively) in PII. As suggested by the percent increase, the most significant change (p < 0.05, Wilcoxon rank sum test) is in mixed-layer Si(OH)_4_ which averaged 0.1 ± 0.11 μg L^−1^ in PI and 1.12 ± 0.63 μg L^−1^ in PII in the mixed layer. ρSi exhibited a similar increase in PII to the macronutrients. Conversely, NPP, new production, the contribution of large phytoplankton to Chl *a*, PC, and PN, and BSi are all higher in PI. There is a large and statistically significant difference (p < 0.05, Wilcoxon rank sum test) in the relative contributions to Chl *a* by size-fractions between PI and PII, where the large size-fraction accounts for 82.3 ± 2.0% in PI but drops to 56.1 ± 7.7% in PII. The substantial differences between phases resulted from a net deepening of the MLD (average of 32.8 m in PI and 42.6 m in PII), which caused horizontal and vertical advection of biomass and an injection of nutrients from below [3]. When assessed with PERMANOVA, the overall environmental parameters differed significantly at the *P* < .1, but not .05, level.

### Phytoplankton succession from diatom-dominated to haptophyte-dominated

**Figure 3:**
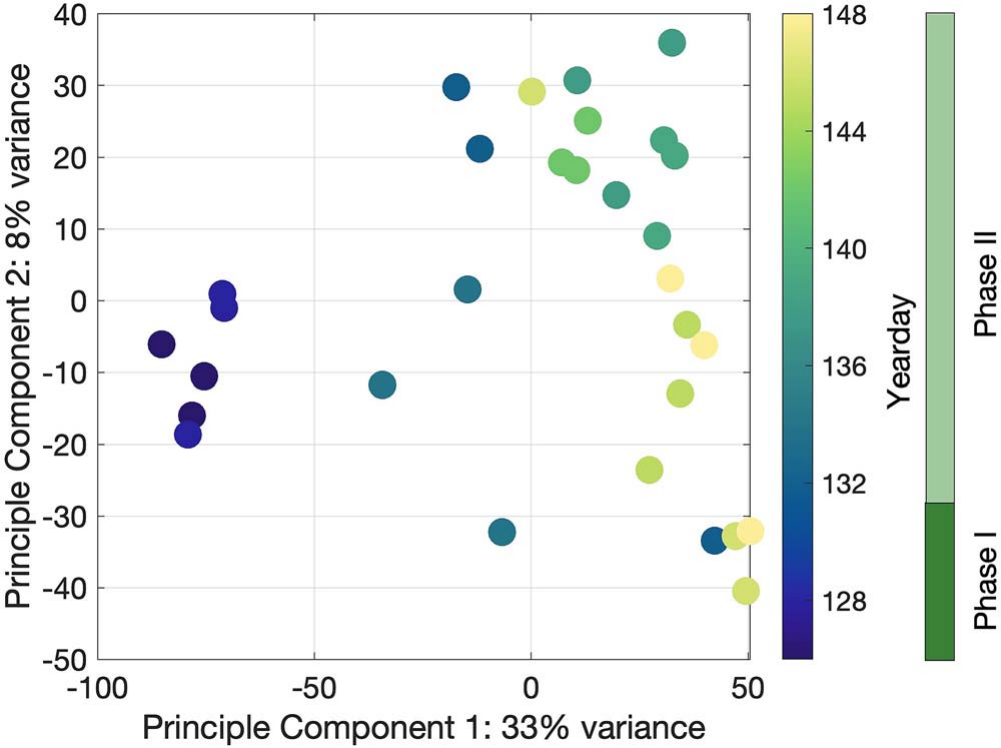
Principal component analysis of metatranscriptomic sequence samples (*n* = 30). Samples are colored by yearday. Vertical green bars indicate phase.

Metatranscriptome sequencing depth ranged from 18.5–33.1 million reads with an average of 27.8 million reads per sample (Table S3). GC content was relatively consistent ranging from 62.0%–68.4% with an average of 65.9% ± 1.64% (Table S3). Statistical analysis of sequenced reads show noteworthy differences in sequence read abundance and taxonomic composition between sampling days grouped into PI versus those in PII (Fig. 3).

**Figure 4:**
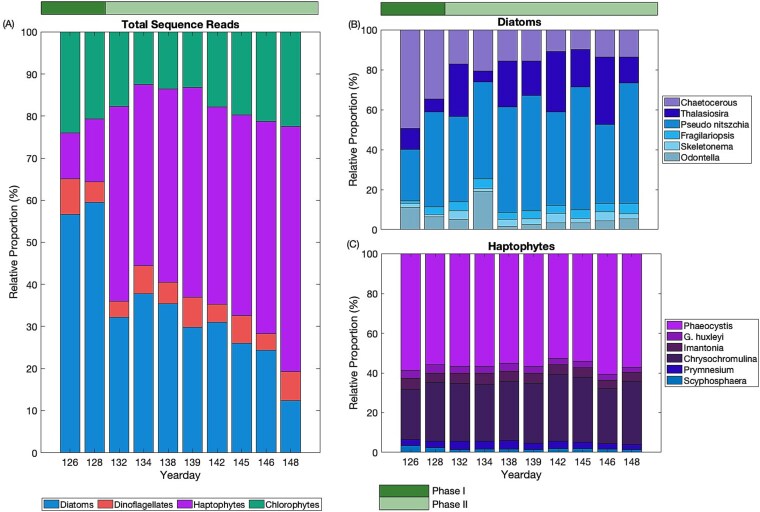
(A) Total relative proportion (%) of the four most abundant phytoplankton groups based on RNA sequence reads, and (B, C) the relative proportion (%) of the most abundant genera in diatoms and haptophytes based on sequence reads. *G. huxleyi* is *Gephyrocapsa huxleyi* (formerly *Emiliania huxleyi*). Horizontal green bars indicate phase.

The total number of reads ranged from 1.85 × 10^7^ on YD 126 to 3.31 × 10^7^ on YD 134 (Fig. 4A). Diatoms constituted the largest percent of reads (~58%) during Phase I, but decreased in Phase II where haptophytes began to constitute the largest relative percentage (46%–58%; Fig. 4A). Both phytoplankton groups exhibited substantial variability with diatoms decreasing 47%, and haptophytes increasing 47% over the observation period. The decline in diatom reads from Phase I to Phase II and the proportional increase in haptophyte contribution to total contigs is noteworthy, supporting both a change in net biomass contribution that coincides with a decline in production as well as a shift in community composition consistent with what was observed in other EXPORTS datasets [16]. Dinoflagellates and chlorophytes remained relatively consistent at ~8% and 18%, of the read proportions, respectively.

### Ecological importance of the diatom *Psuedo-nitzchia* and the haptophyte *Phaeocystis*

Within the diatoms, members of *Pseudo-nitszchia* (48%), *Chaetocerous* (20%), and *Thalassiosira* (18%) were the three most represented genera based on RNA sequence reads (Fig. 4B). The haptophytes had a similar predominance of one main genera with *Phaeocystis* accounting for 56% of total reads and *Chrysochromulina* and *Prymnesium* accounting for 30% and 4%, respectively (Fig. 4C; see Supplemental Results S2.1 for dinoflagellate and chlorophyte representation).

### Temporal differences in diatom and haptophyte physiological activity

**Figure 5:**
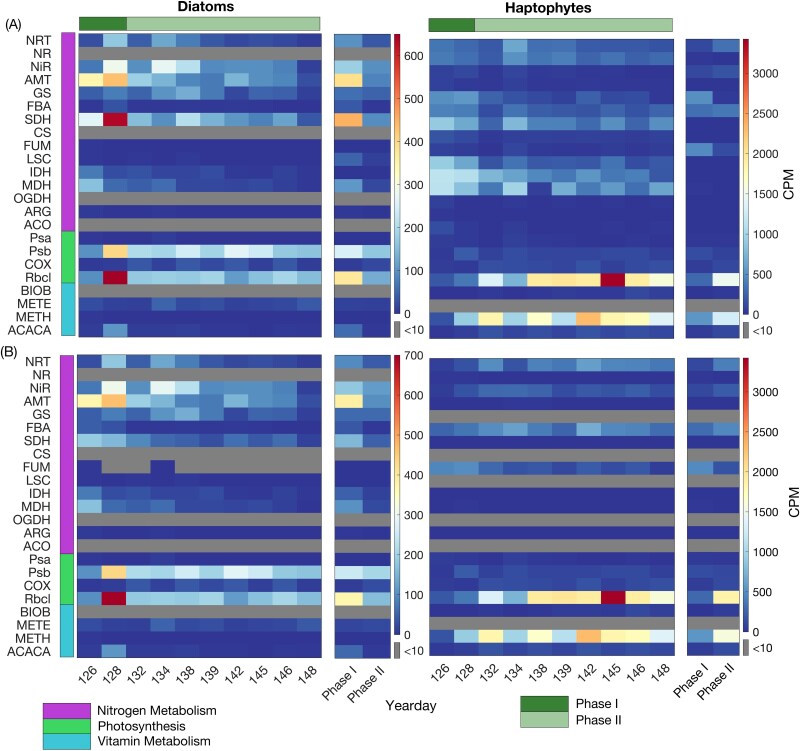
Heatmaps of transcript CPMs of key nitrogen metabolism, cell maintenance, photosynthesis, and vitamin metabolism genes (indicated by left hand bars) for diatoms and haptophytes with counts normalized within each taxonomic group (A) and to the whole community (B). Plots are by yearday and phase average (normalized by the number of sample days) with the color bar indicating CPM for each gene. Horizontal green bars indicate phase. Full gene names are: nitrate transporter (*NRT*), nitrate reductase (*NR*), nitrite transporter (*NiR*), ammonium transporter (*AMT*), glutamine synthase (*GS*), flavodoxin (*FBA*), succinate dehydrogenase (*SDH*), citrate synthase (*CS*), fumarate (*FUM)*, succinyl-CoA synthetase (*LSC*), isocitrate dehydrogenase (*IDH*), malate dehydrogenase (*MDH*), oxoglutarate dehydrogenase (*OGDH*), arginase (*ARG*), aconitase (*ACO*), photosystem I (*Psa*), photosystem II (*Psb*), cytochrome c oxidase (*COX*), RUBisCO (*Rbcl*), biotin synthase (*BIOB*), methionine synthase (*METE*), methyltransferase (*METH*), and acetyl-CoA carboxylase (*ACACA*).

The transcript abundance of key genes can be categorically separated into those relating to nitrogen metabolism, photosynthesis, vitamin metabolism, and for diatoms, silicon metabolism. Reads are presented in two ways: normalized both within phytoplankton groups (Fig. 5A) and to the whole community (Fig. 5B). Normalizing by whole community transcripts provides a comparative analysis on how the proportions of each metabolic activity are changing in each phytoplankton group relative to the other groups whereas the taxon-specific normalization provides an estimate of changes in gene expression within a phytoplankton group. Despite substantial variability amongst phytoplankton groups, distinct patterns are visible between how the expression of various genes changed through time, particularly between PI and PII.

**Figure 6:**
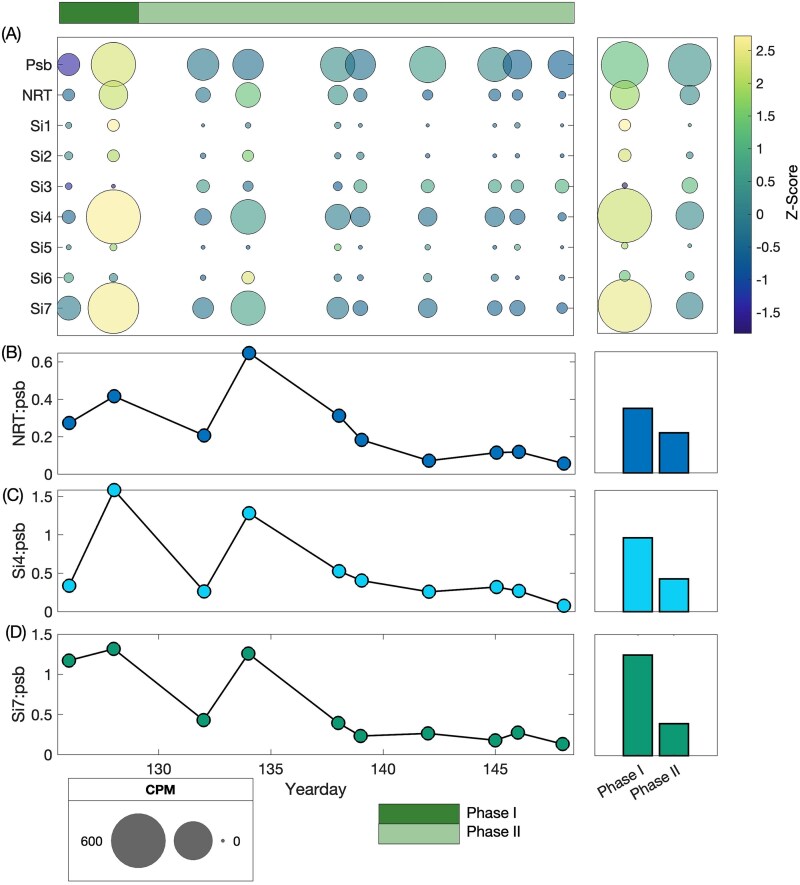
(A) Transcript CPMs of diatom *Psb, NRT,* and silicon transport genes normalized within the diatom group by yearday and phase average normalized to the number of sample days and ratios of diatom (B) *NRT:Psb*, (C) Si4:*Psb*, and (D) Si7:*Psb* by yearday and phase average. The size of the symbol in panel (A) corresponds to CPM. The color bar indicates the *Z*-score. Further information on the Si gene4 and Si gene7, both of which are SITs, is available in table S2. Green bars indicate phase.

Diatoms displayed the highest expression of all genes on YD 128 where photosynthesis-related genes exhibited their maximum transcript abundances of the entire observation period. Within the diatom-specific normalization, genes encoding for photosynthetic proteins, RubisCO (*Rbcl*) and photosystem II (*Psb*), and nitrogen assimilation proteins, ammonium transporter (*AMT*; p-value <0.05), nitrite reductase (*NiR*), and acetyl-CoA carboxylase (*ACACA*) increased 5.8-, 3.1-, 0.2-, 3.1-, and 9.4-fold change in PI from YD 126 to YD 128, respectively (Fig. 5A). While changes in all genes are large, the changes in expression of ammonium transporters proved the most significant (Fig. S3). The changes in counts normalized to the entire community are comparable (Fig. 5B). Of the seven diatom-specific silicon transport genes analyzed, two (Si4 and Si7) showed similar trends to the photosynthesis related genes, peaking on YD 128 (Fig. 6B and C). Not all patterns were statistically significant with results likely impacted by the uneven distribution of sampling days between phases. These genes are annotated as silicon transporters from *Fragilariopsis cylindrus* and *Phaeodactylum tricornutum*, respectively (Table S4; [35, 36]). YD 128 coincides with the highest number of sequences of the four primary taxa for any sample day as well as the highest proportional contribution of diatom sequences to total sequences (35.9%; Fig. 4A). During PII, transcripts of *Psb, Rbcl*, and *NiR* were on average higher than on YD 126, a noteworthy distinction, suggesting that despite lowered rates, NPP and new production still occurred (Figs 2, 5A and B). The second highest counts for Si4 and Si7 are also observed in PII on YD 134 (Fig. 6B and C), which coincides with the beginning of the second large storm event and an increase in Si(OH)_4_ concentrations in the mixed layer [3].

Haptophytes exhibit the most appreciable difference between gene expression patterns normalized at the phytoplankton group versus whole community level of any of the examined taxa (Fig. 5A and B). This is apparent from numerous genes involved in cell maintenance and nitrogen metabolism which are better represented in the haptophyte specific normalization but are weakly expressed (CPM <10) in the community normalization, including glutamine synthase (GS), citrate synthase (CS), isocitrate dehydrogenase (IDH), oxoglutarate dehydrogenase (OGDH), and aconitase (ACO) (Fig. 5B). However, some consistent patterns still emerge between the two approaches and suggest substantial differences exist between PI and PII, particularly in terms of photosynthesis. In the haptophyte-specific normalization, *Rbcl* and *METH* (both *P*-value <.05) are on average 5.8- and 2.0-fold higher in PII with similar patterns for the community normalization. While the differences between phases are not as obvious, *NRT* also increased substantially (>1400%; *P*-value <.05) between PI and PII. Across both PI and PII, *Psb* exhibited higher abundances than *Psa*, suggesting that like in diatoms, haptophytes may be engaging in new production with a preference for expression of *Psb* (Fig. 5A, 5B). Similarly to diatoms, these changes between phases coincide with shifts in the relative proportion of haptophyte to total sequences (Fig. 4B), but it is noteworthy that photosynthesis and vitamin metabolism genes in haptophytes exhibit significant temporal patterns but in diatoms, these gene expression patterns are not significant (Fig. S3). Contrastingly, gene expression patterns in dinoflagellates and chlorophytes were consistent across phases (see Supplemental Results 2.2).

### Si-related gene expression suggests decreased silicic acid uptake and primary production

To assess how changes in gene expression drove changes in NPP, new production, and *ρ*Si, we calculated numerous ratios of key genes for overall primary production and diatom cell growth, including *NRT*:*Psb* and Si:*Psb* (Figs 6;  S6). For diatoms, the Si-related gene ratios were assessed and compared to ^32^Si-*ρ*Si rates obtained during the observation period. Ratios of Si4:*Psb*, Si7:*Psb*, Si4:*NRT*, and Si7:*NRT* were all higher in PI relative to PII, but Si7:NRT exhibited the only statistically significant increase (*P*-value <.05; Fig. 6B and C). This pattern may suggest that the Si7 transporter is more sensitive to differences between silicic acid and nitrogen metabolism than Si4 transporter is. During PI, ratios of Si7:*Psb* and Si7:*NRT* were overall higher than ratios of Si4:*Psb* and Si4:*NRT* despite the Si4 transcript abundance being higher (137 CPMs) and exhibited more temporal variability (± 187) than Si7 (average = 129 ± 154). When evaluating *ρ*Si:*ρ*DIC vs. Si4(7):*Psb* and *ρ*Si:*ρ*NO_3_^−^ vs. Si4(7):*NRT* using a principle component analysis, the strongest relationships were between large size-fraction *ρ*Si:*ρ*DIC vs. Si7:*Psb* (*R*^2^ = 0.65) and large size-fraction *ρ*Si:*ρ*NO_3_^−^ vs. Si7:*NRT* (*R*^2^ = 0.51). Both relationships were negative (Fig. S6), and multiple linear regression analysis suggests uptake and Si7 gene expression patterns are influenced by environmental conditions in the mixed layer (e.g. temperature and PAR; Table S5).

## Discussion

### Spring bloom phenology

The annual North Atlantic spring bloom is known to exhibit a predictable taxonomic succession from diatom to haptophyte dominance coincident with a decline in nutrient concentrations, particularly NO_3_^−^, and resulting phytoplankton biomass [7, 16]. Here, our findings suggest that the 2021 bloom exhibited a typical taxonomic succession associated with bloom decline. However, instead of NO_3_^−^ limitation, Si(OH)_4_ depletion in Phase I may have created an unexpected physiological response in diatoms that hindered primary production yet resulted in enhanced pulses of carbon export [37]. Our transcriptomic results support this finding, suggesting this physiological response persists into Phase II when Si(OH)_4_ concentrations were partly replenished with storm-induced mixing. Silicic acid limitation of diatoms was confirmed by Si addition incubation experiments [22], but the inability for diatoms to recover physiologically in the days to weeks following natural Si(OH)_4_ replenishment is noteworthy and contrary to previous findings [38]. Combined with gene expression trends, this suggests a decoupling between intracellular activity and ecosystem rate measurements due to substantial reductions in the biomass of large cells such as diatoms, likely caused by loss processes such as grazing and export and negative physiological impacts caused by depleted silicic acid concentrations during PI.

As shown through the transcript abundance plots (Figs 5 and 6), our analysis suggests strong temporal differences between the physiological patterns amongst the different phytoplankton groups, particularly diatoms and haptophytes. For diatoms, YD 128 clearly represents a transition where the decoupling between the transcription of key metabolic genes (*Rbcl, Psb, AMT*, and *NRT*), NPP and new production rates is most pronounced. YD 128 marks the peak in photosynthesis gene expression, but this does not appear to translate into higher NPP or new production. For haptophytes, YD 126 was the most distinct (Fig. S4; See Supplemental Methods S1.1. and Results S2.3.) and represents a day of lowered expression of genes related to new production (*NRT*:*Psb* is 1.64; Fig. S6). For haptophytes, YD 126 marks the lowest transcription of *Psb* and *METH* with 20 and 755 fewer transcripts relative to peak values of 210 and 2302 on YD 128 and 142, respectively. Both the diatoms and haptophytes, their day with the largest difference in transcript abundances occurs in PI. This reinforces the observation that the transition from PI to PII marks a substantial shift in the biogeochemical and taxonomic attributes of the system coincident with the first mixing event.

### Phytoplankton succession during bloom decline

To better understand the drivers of these taxonomic and temporal differences, key phase dynamics were examined, paying particular attention to changes in the Si-related genes relative to changes in better understood metabolic genes, such as *Psb, NRT*, etc. The two most expressed Si genes, Si4 and Si7, have strong similarity to known silicic acid transporters (SITs) in the NCBI repository (Table S2; [34, 37]). However, there is still a general lack of understanding of SIT activity and silica dynamics in diatoms, partially due to the poorly conserved nature of SITs, which allows them to quickly adapt to changes in their environment [39]. Gene expression analysis has shown that some SITs cluster together, suggesting coordinated activity [40]. The similarity of patterns in Si4 and Si7 may be an example of this type of clustering. Whether these SITs can act independently or require co-expression is uncertain.

Previous studies have shown how SIT activity can be inversely related to Si(OH)_4_ concentration [41, 42] but is closely related to the cell cycle [39]. While some uncertainty exists around the subject [43], enhanced expression of SITs without a congruent increase in primary production may support the idea that this could lead to enhanced silicification, creating heavier ballasted diatom cells that may facilitate faster sinking and enhanced carbon export out of the euphotic zone [44]. This is consistent with optical and sediment trap data, which indicated that more large particles were exported from the upper water column and a higher representation of biogenic silica in trap material in the latter part of the observation period [22, 45].

While overall nutrient uptake rates in PI were driven by diatoms, these dynamics appear driven mostly by non-diatoms in PII, particularly haptophytes. Despite having fewer overall transcripts, due to smaller genomes in some species [46], haptophytes may be a key contributor to primary production in PII of the observation period when rates of NPP and new production have declined (Fig. 2; Table S5). During this time, haptophytes maintain substantial transcription of key genes for photosynthesis (*Rbcl*) and methionine synthase (*METH*) as well as a high (average = 4.77 ± 1.89) ratio of *NRT*:*Psb*, potentially suggesting utilization of NO_3_^−^ (Fig. 5A, 5B;  Fig.  6A) [47], consistent with the idea of reduced resource competition. This idea could contradict the previously proposed rising tide hypothesis (i.e. conditions favorable for diatoms will be likewise favorable for other phytoplankton groups, and vice versa; [48]) and suggest that resource competition may be lowered during this time as diatoms are less metabolically active. The high proportion of *Phaeocystis*, which contains a nitrogen rich colonial fluid [49, 50], is noteworthy and raises the question of potential luxury storage of nutrients and competitive advantage.

In our two less dominant phytoplankton groups, dinoflagellates and chlorophytes, we do not see as strong gene expression trends as in diatoms and haptophytes. One exception to this is *Psb* in dinoflagellates, which substantially increases in abundance from PI to PII (Fig. S2A;  S2B). This increase coincides with a substantial decrease in mixed layer biomass (46.6% in Chl *a* and 23.4% in PC) and may signify a shift toward greater autotrophy within the dinoflagellates. Additionally, the dinoflagellate community composition does not appear to change substantially (Fig. S1A), perhaps suggesting the shift may result from mixotrophic capabilities rather than a shift in the taxonomy [51]. This notion is further supported by the finding that the three most transcriptionally active dinoflagellate genera, *Karlodinium, Alexandrium,* and *Karenia*, are all known to be mixotrophic [52–54]. As a much smaller proportion of the phytoplankton community, chlorophytes’ key metabolic genes, including *Rbcl, Psb*, NRT, AMT, etc., were not detected and thus, do not appear to impact net biogeochemical trends substantially (Figs S2A;  2B).

### Biogeochemical implications

Concerted research efforts have gone into understanding how Si availability impacts diatom metabolism. It is well established that low Si concentrations limit cell growth and reproduction which can then limit the extent of diatom blooms [31], but Si as a limiting factor or co-limiting factor for primary production is less understood [43]. Maniscalco *et al.* [43] found that rather than Si stress alone inhibiting primary production, it can be co-limiting with N when both N and Si are depleted. In our case, NO_3_^−^ concentrations remained >4 μM and were not considered limiting to growth, potentially suggesting Si alone can independently limit diatom primary production. Concurrent measurements during the EXPORTS cruise did not find a strong response by diatoms to NO_3_^−^ addition, further suggesting nitrate was not independently limiting [55]. Given the key contribution of diatom production to total rates in this system [16], the extremely low Si(OH)_4_ concentrations (average of 1.0 ± 0.5 μmol l^−1^) and abundance of light and other nutrients suggests Si availability was the primary control on diatom NPP during PI. Our gene expression data supports this physiological observation with variable silicon transporter expression corresponding to changes in ambient silicic acid concentrations. Recent studies have found that Si limitation in diatoms can lead to increases in viral infection and subsequently more remineralization and less carbon export [56]. Our diatom gene expression data did not exhibit varying expression of genes associated with viral infection, but a more targeted analysis of viral infection and lysis and the impacts on carbon cycling in the North Atlantic is warranted.

During the decline phase of the NA spring bloom, there was a transition from a diatom-dominated community to one dominated by other, non-diatom phytoplankton taxa. These other phytoplankton groups (particularly smaller-celled groups such as haptophytes) can often be responsible for a majority of the primary production and nutrient cycling in various regions both within and outside of bloom conditions [16, 57, 58]. Our results support previous findings [59], which suggest that as diatoms decline upon Si(OH)_4_ depletion in PI, haptophytes capitalize on reduced resource competition and increase their gene expression related to new production. This creates more balanced contributions of small (i.e. non-diatom) and large (i.e. diatoms) cells to total community uptake trends in PII. However, the role of diatoms in NPP trends is still important, as suggested by the strongest relationship between diatom *NRT*:*Psb* and NPP by the large size-fraction across both PI and PII (*R*^2^ = 0.63; Fig. 6A). On the other hand, dinoflagellate and haptophyte trends between *NRT*:*Psb* (Fig. S5; See Supplemental Results S2.4.) and NPP by the large size-fraction are slightly lower at *R*^2^ = 0.62 and 0.53, respectively. While this trend is likely specific to Si(OH)_4_ limiting conditions, as it will disproportionally limit diatom growth and productivity, it represents larger ecosystem dynamics with diatoms appearing to respond more readily to changes in environmental conditions relative to haptophytes and dinoflagellates. Interestingly, there was a strong and significant positive relationship between the relative proportion of diatoms and haptophytes in the surface community and sediment traps [60], further supporting the notion that these two phytoplankton groups are disproportionately important to carbon export and biogeochemical cycling. Our analysis supports the ecological and biogeochemical importance of diatoms and haptophytes as observed through congruent patterns between community-wide rates and phytoplankton group-specific gene expression.

Our findings suggest frequent storms throughout the spring in the North Atlantic can have a profound influence on spring bloom dynamics, community composition, and phenology. Uncertainty exists regarding how climate change will impact storm events in the North Atlantic [61], making it difficult to assess whether the findings presented here are exceptions or will become more frequent in the coming years. Understanding how different phytoplankton groups respond to these storm events, particularly in terms of variable mixed layers and nutrient concentrations during the bloom, and how the interplay of resource competition impacts ecosystem biogeochemistry is critical to understanding carbon export potential and future climate change in the region.

## Conclusions

Our study provides new insights into some of the molecular underpinnings that control bloom dynamics in this climatologically important region and suggests that diatoms and haptophytes are dominant drivers of primary production in this system. High rates of primary production, primarily diatom-driven, appear to have controlled Si(OH)_4_ depletion during the North Atlantic annual spring bloom, resulting in sustained Si(OH)_4_ limitation in the diatoms. As the bloom declined, Si(OH)_4_ depletion creates a decoupling between silicon metabolism from community carbon and nitrogen metabolism, as shown through the activity of two key silicon transporters relative to key photosynthesis (*Psb, Rbcl*) and nitrogen metabolism genes (*NRT, AMT*). Due to their lack of silicon requirement, haptophytes also appeared to metabolically respond to decreased diatom productivity and apparently reduced competition for resources during the second phase. Diatoms remain present in the system and are responsible for net ecosystem dynamics, particularly carbon export, despite the increased contribution of haptophytes during bloom decline. These findings support previous studies, which suggest diatoms are responsible for net bloom dynamics while highlighting the key role haptophytes play during bloom succession over time.

## Supplementary Material

Meyer_Chapter3_Supplemental_v3_ycag176

## Data accessibility

All data presented here are available at the SeaWiFS Bio-optical Archive and Storage System (SEABASS; seabass.gsfc.nasa.gov/cruise/EXPORTSNA) or at the National Center for Biotechnology Information (NCBI; ascension number PRJNA1072555). RNA analysis code is available at [32].
